# Social media, internet use and suicide attempts in adolescents

**DOI:** 10.1097/YCO.0000000000000547

**Published:** 2019-07-22

**Authors:** Rosemary Sedgwick, Sophie Epstein, Rina Dutta, Dennis Ougrin

**Affiliations:** a Department of Child and Adolescent Psychiatry, Institute of Psychiatry, Psychology and Neuroscience, King's College London; b South London and Maudsley NHS Foundation Trust; c Department of Psychological Medicine, Institute of Psychiatry, Psychology and Neuroscience, King's College London, London, UK

**Keywords:** adolescents, internet, social media, social networking sites, suicide, suicide attempt

## Abstract

Suicide is the second leading cause of death in youth aged 10–24 years old globally, but detecting those at risk is challenging. Novel preventive strategies with wide influence across populations are required. Interest in the potential for both detrimental and supportive influences of social media/internet use on suicidal behaviour has been growing; however, the relationship remains unclear.

**Recent findings:**

A systematic search of articles from database inception up to 25 January 2019 across five databases: Medline, PsycINFO, EMBASE, HMIC and CINAHL revealed nine independent studies investigating social media/internet use and suicide attempts in young people less than 19 years old (*n* = 346 416). An independent direct association was found between heavy social media/internet use and increased suicide attempts in seven studies (adjusted ORs ranged from 1.03 to 5.10), although adjusting for cyberbullying victimization and sleep disturbance reduced the strength of this association. Two studies found that some social media/internet use, versus no use, may be associated with fewer suicide attempts. There were no studies investigating the relationship between social media/internet use and completed suicide.

**Summary:**

There is an independent association between problematic use of social media/internet and suicide attempts in young people. However, the direction of causality, if any, remains unclear. Further evaluation through longitudinal studies is needed.

## INTRODUCTION

Suicide is the second leading cause of death in young people aged 10–24 years, globally [1]. However, it is challenging to detect and intervene early, as many of those who go on to die by suicide will not have interacted with mental health services [2]. Novel mechanisms that underpin suicidal behaviours are required.

The internet is now ubiquitous globally and is used for educational, recreational and social purposes. Social network sites (SNSs) and social media are web-based services entitling users to construct a personal profile, support user-generated content, connect with other users and support ways for members to collaborate [3,4]. However, as technology has progressed, the boundaries between internet, social networking sites (SNSs), social media, online gaming and digital technology have become increasingly blurred.

The links between self-harm, suicidal ideation and later suicide attempts are well established [5,6], but nomenclature in this field varies. In the United States, suicide attempts, nonsuicidal self-injury (NSSI) and self-harm (with undetermined intent) are described separately, in contrast to Europe where ‘self-harm’ is used more broadly [7–9]. We will focus on suicide attempts, one of the strongest known predictors of completed suicide, and will specifically focus on the evidence pertaining to adolescents aged 18 years and under, to be relevant to child and adolescent mental health service provision in most countries.

Seven systematic reviews published to date have found an association between increased screen time and worse mental health in young people [10^▪▪^], and the association between cyberbullying and suicidal behaviour is described in a recent meta-analysis [11^▪▪^]. However, the existing evidence suggests that the relationship between internet use and self-harm and suicidal behaviour is mixed [12^▪▪^] with potential for harm, but also scope to foster a sense of community, offering isolated young people supportive contacts [13]. Guidance is required, but there remains a paucity of evidence to inform this. There are suggestions for strategies, such as: a ‘Family Media Use Plan’ [14], ‘Digital Literacy’ being taught in schools [15,16], increased support for parents [16] and the need for funding of new research, to ensure future guidance is evidence-based [17^▪^].

No previous review has specifically investigated the association between social media/internet use and completed or attempted suicide in adolescents (under 19 years). This review aims to fill that knowledge gap, as well as outline some of the recent developments in this field of enquiry. 

**Box 1:**
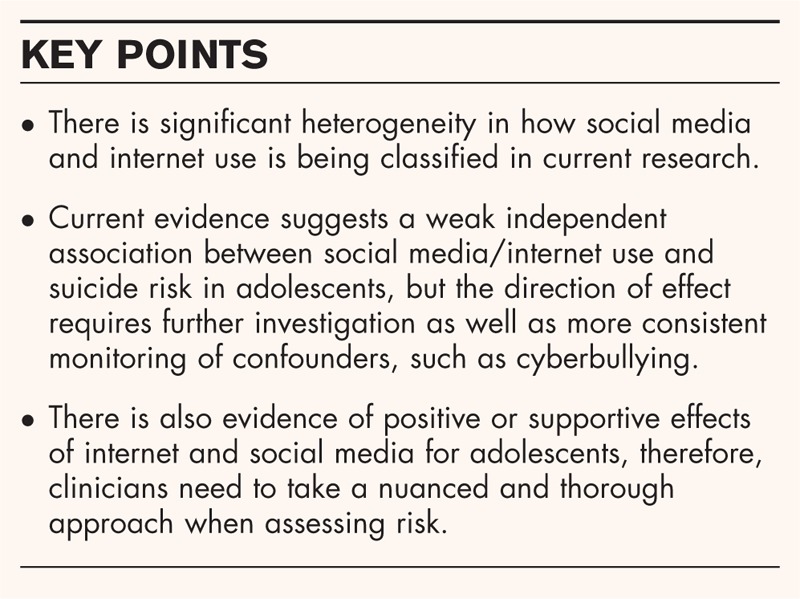
no caption available

## METHOD

This review is reported according to the Preferred Reporting Items for Systematic Reviews and Meta-analyses (PRISMA) guidelines and the protocol is registered on PROSPERO (ID CRD42018115259). English language publications, published from database inception up to 25 January 2019 were included. Studies with a majority of participants under 19 years old at the point of enrolment in the study were included.

We were interested in the following exposures: patterns and nature of social media and internet use, and content viewed or shared online. Due to the high prevalence of social media/internet use in young people, studies comparing different types, or levels, of internet or social media use were included. Only peer reviewed, observational studies, where the full text was available were included. Qualitative studies, case reports or comments/editorials were excluded.

The outcome of interest was suicide or suicide attempts in children and adolescents. Due to the practical challenges of determining intent from the wider ‘self-harm’ literature, only studies specifically stating ‘suicide attempts’ as their outcome were included. NSSI and self-harm, without suicidal intent or where intent was not specified, were not included.

### Literature search

The following databases were searched with a predefined search strategy: Medline, PsycINFO, EMBASE, HMIC and CINAHL. The search strategies were developed and adapted to include both subject headings (i.e. Child, Adolescent, Social Media, Internet, Suicide, Suicide Attempt) and keywords, that is, ‘Facebook’, ‘Instagram’, ‘hashtag∗’, ‘suicid∗’ relevant to each respective database. The full search strategies for each database are available in the online supplement. In addition, backward and forward citation searching was conducted and the reference lists of existing systematic reviews on similar topics were reviewed to identify any further relevant articles.

### Data extraction and quality assessment

Abstract screening, data extraction and quality assessment of articles was completed by two independent reviewers (R.S. and S.E.). Full text articles were obtained and screened by the two reviewers where suitability could not be determined based on the title and abstract. Data was extracted using a predesigned data extraction form. Risk of bias was assessed using an adapted Newcastle-Ottawa scale (see supplementary material). Scores of 0–4 were considered as high quality (low risk of bias), 5–7 as moderate quality and 8–10 as low quality (high risk of bias).

## RESULTS

**FIGURE 1:**
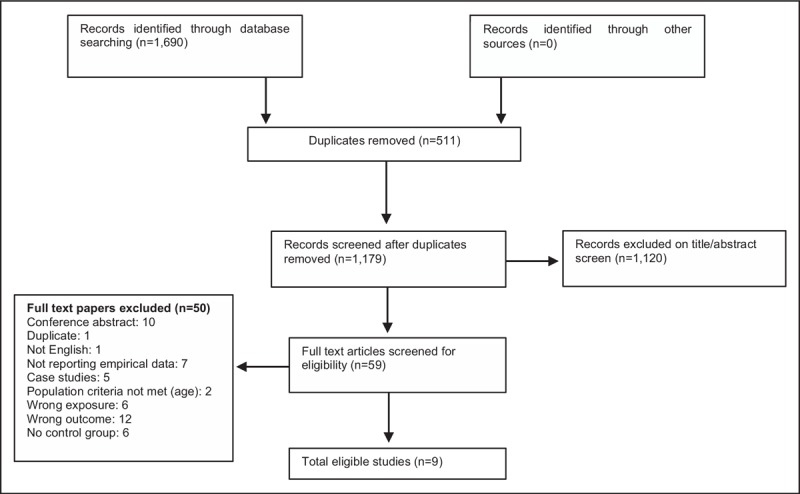
PRISMA flow diagram. The databases searched were: Medline, PsycINFO, EMBASE, HMIC and CINAHL.

One thousand six hundred and ninety references were identified through database searching and 1179 remained after removing duplicates. One thousand one hundred and twenty were excluded on title and abstract screen. Fifty-nine full texts were reviewed of which nine were eligible for inclusion in the review. Figure 1 (PRISMA diagram) shows further details, including reason for exclusion at the full text screening stage.

Of the nine identified studies, all were cross-sectional, published between 2012 and 2018. The age range of participants studied was 11–18 years. In five of the studies, the female participant rate was over 50%, most studies had an approximately equal proportion of male and female participants. Studies were conducted in a range of countries with sample sizes from *n* = 111 [18^▪^] to *n* = 221 265 [19]. There were a total of 346 416 participants across the nine studies. Six articles were judged to be of high quality, two of medium quality and one low quality. The two medium-quality studies lacked information on nonrespondents and appropriate statistical tests. Ascertainment of the exposures and outcomes within each article showed low risk of bias, but there was significant heterogeneity between studies.

One study investigated psychiatric outpatients with Major Depressive Disorders [20], another a psychiatric inpatient setting [18^▪^], the rest were community-based or school-based samples. Two studies examined internet use as the outcome [21,22], the remainder analysed suicide attempts as the dependent outcome. Control groups in those with social media or internet use as the exposure were either lower level internet users [19], nonusers [23], occasional users [24] or those not meeting the defined threshold for pathological [18^▪^], problematic [18^▪^,^22^,^25^^▪▪^] or addicted [26] internet behaviours.

There was significant heterogeneity in both the method of assessing social media/internet use and the timescale of reported suicide attempts. Only one study investigated SNSs. Problematic Internet Use was the exposure of interest, classified as a score on the Young Internet Addiction Test (YIAT) of over 50, in two studies [20,25^▪▪^]. In another, Pathological Internet Use, defined as at least 5 on the Young's Diagnostic Questionnaire (YDQ) was the outcome [21]. Lin *et al.* used the addiction cut-off score of 64 on the Chen Internet Addiction scale to define their exposure. The remaining studies were interested in hours of noneducational internet use [24] and cut-offs on the Korean Internet Addiction Self-Assessment Tool [19].

There was an association between increased social media/internet use and suicide attempts in seven studies when controlling for at least age and sex [adjusted odd ratios (ORs) ranged from 1.03 to 5.10] [23,25^▪▪^]. Alpaslan *et al.*[20] found no relationship between suicide attempts and YIAT score while controlling for age and sex in Major Depressive Disorder cases and Fuchs *et al.*[18^▪^] did not report any multivariable results. The association between Social Networking Sites (SNS) and suicide attempts found by Sampassa-Kanyinga (OR 5.10, 95% CI 1.45–17.88) was found to be indirect and explained by cyberbullying victimization. Guo *et al.*[25^▪▪^] found that on path analysis, sleep disturbance was found to be a mediator in the link between Problematic Internet Use and suicide attempts. Cyberbullying victimization and sleep disturbance were not controlled for in other studies.

Kim [24] found both male and female ‘heavy internet users’ showed an increased attempted suicide rate compared with ‘no internet users’, but ‘occasional internet users’ had the lowest rate of attempted suicide. Similarly, Lee *et al.* found that the Korean Internet Addiction Self-Assessment Tool (KS scale) determined a ‘high risk’ of internet addiction user group (OR 1.91, 95% CI 1.71--2.14), and the ‘nonuser’ group (OR 1.33, 95% CI 1.25--1.42) were more likely to attempt suicide than the ‘potential-risk’ group (OR 1.20, 95% CI 1.04--1.38). These results indicate that some degree of internet use may be beneficial.

Unfortunately, because of the heterogeneity in exposures and outcomes, results could not be combined in meta-analysis to provide a meaningful result; however, all nine studies are summarized in Table 1.

**Table 1:** Summary of included studies

Lead author, year, country	Study design features	Study population	Exposure	Outcome	Key findings	Quality rating
Alpaslan, 2016, Turkey	Cross sectional study exploring associations between PIU and suicide attempts among patients with Major Depressive Disorders (MDD)	*N* = 120, 12--18-year-old outpatients with MDD, mean age 15.22 years	Potential PIU defined as a YIAT score at least 50	Self-reported suicide attempts (last 6 months)	No significant difference in PIU rates between patients with MDD who had, and had not, attempted suicide (*χ*^2^ 2.35, *P* = 0.188). No relationship between suicide attempts and the YIAT score, controlling for age and sex	7
Fuchs, 2018, Austria	Cross sectional study assessing correlations between PIU and psychiatric comorbidities in adolescent inpatients	*N* = 111, 12--17-year-old inpatients, mean age 15.1 years	CIUS at least 21 for PIU; at least 28 for pathological or addictive internet use	“History of suicide attempt’	Patients with PIU showed significantly more suicide attempts than patients without (*χ*^2^ 13.78, *P* < 0.001)	4
Kaess, 2014, Europe	Cross sectional study investigating associations between Pathological Internet Use and self-destructive behaviours	*N* = 11 356 school-based adolescents, mean age 14.9 years (range not reported)	Self-reported suicide attempt (lifetime)	Pathological Internet Use, YDQ score at least 5	Suicide attempts were positively associated with Pathological Internet Use in multilevel mixed-effects linear regression (coefficient 0.530, 95% CI 0.185–0.875, *P* = 0.003) and stepwise regression (coefficient 0.552, 95% CI 0.207-- 0.896, *P* = 0.002).	9
Kim, 2012, Korea	Cross sectional study investigating associations between noneducational purpose internet use-time and health status	*N* = 75 066 school-based adolescents, grades 7--12	Noninternet users (NIUs -- no use last 30 days), occasional internet users (OIUs – less than 1 h/day), moderate internet users (MIUs >1 h, < 2h/day), heavy Internet users (HIUs >2 h/day)	“Experience rate of attempted suicide’	OIUs were the largest group, with lowest rates of attempted suicide. Multivariable results with OIU as reference. Girls: NIU (OR 1.23, 95% CI 1.05-- 1.43), MIU (OR 1.37, 95% CI 1.18–1.60), HIU (OR 2.04, 95% CI 1.41–2.95). Boys: NIU (OR 1.87, 95% CI 1.61--2.18), MIU (OR 1.51, 95% CI 1.20–1.90), HIU (OR 3.41, 95% CI 2.43–4.79)	8
Lee, 2016, Korea	Cross sectional study investigating associations between level of internet addiction and suicide attempts in adolescents	*N* = 221 265 middle and high school students	Level of internet use: hours in last 30 days, with a modified KS scale. KS scale cut-offs: mild user (≤ 47), potential risk for addiction (48–52) and high risk for addiction (≥53)	Self-reported suicide attempt (last 12 months)	With mild users as the reference group, the high-risk user group were most likely to attempt suicide (OR 1.91, 95% CI 1.71--2.14); but the nonuser group (OR 1.33, 95% CI 1.25--1.42) was more likely to attempt suicide than the potential-risk group (OR 1.20, 95% CI 1.04--1.38)	8
Lin, 2014, Taiwan	Cross sectional study investigating associations between suicide attempts, internet addiction and various internet activities	*N* = 9510 adolescent students aged 12–18 years	Internet addiction and activities- CIAS of at least 64 or more	Self-reported suicide attempt (lifetime)	Internet addiction was associated with suicide attempts (OR 1.59, 95% CI 1.29–1.96, *P* < 0.001). Risk of suicide attempts were increased with Internet ‘Chatting’ (OR 1.34, 95% CI 1.10–1.63, *P* = 0.003), online gaming (OR 1.31, 95% CI 1.05–1.63, *P* = 0.017), watching movies (OR 1.26, 95% CI 1.03–1.53, *P* = 0.025), shopping (OR 1.48, 95% CI 1.15–1.90, *P* = 0.002), gambling online (OR 2.43, 95% CI 1.38–4.30, *P* = 0.002) and decreased watching news online (OR 0.73, 95% CI 0.54–0.98, *P* = 0.038).	9
Rikkers, 2016, Australia	Cross sectional study describing associations between Problematic Internet Use/electronic gaming behaviour and risk taking behaviours	*N* = 2967, community-based 11–17-year-olds. Results for suicide 13–17 years only	Self-reported suicide attempt (last 12 months)	Four out of five problem behaviours related to internet use/electronic gaming on the adapted EU Kids Online Survey	Attempted suicide was associated with Internet/electronic gaming ’Problem behaviour’ in Univariate analysis (OR 7.5 95% CI 4.2–13.6) and Multivariate analysis (OR 3.0 95% CI 1.5–6.2).	7
Sampassa Kanyinga, 2015, Canada	Cross sectional study investigating associations between Social Networking Sites (SNSs) use, suicide attempts, and the potential mediating role of cyberbullying victimization	*N* = 5126, school based 11–20-year olds (mean age 15.2 years)	Regular or daily SNSs users	Self-reported suicide attempt (last 12 months)	Use of SNSs was associated with suicide attempts (OR 5.10, 95% CI 1.45–17.88) in binary logistic regression, but this association was explained by cyberbullying victimization in hierarchical logistic regression analysis	8
Guo, 2018, China	Cross sectional study investigating associations between PIU and suicidal behaviour and whether sleep disturbance is a mediating factor	*N* = 20 895 students in grades 7–12	PIU, assessed with the Chinese YIAT. Moderately addicted 50–79, severely addicted 80–100 points	Self-reported suicide attempt (last 12 months)	Severely addicted internet users had greater risk of suicide attempt (14.0%) than moderately addicted (4.3%) and average (1.5%) internet users. PIU was associated with suicide attempts, (adjusted OR 1.03, 95% CI 1.02–1.04, *P* < 0.5) on multivariable, multilevel logistic regression, but sleep disturbance partially mediates this association (standardized *β* estimate = 0.082, 95% CI = 0.068–0.096, *P* < 0.05).	9

CIAS, Chen Internet Addiction Scale; CIUS, Compulsive Internet Use Scale; KS, Korean Internet Addiction Self-assessment Tool; PIU, Problematic Internet Use. Pathological Internet Use is not abbreviated; YDQ, Young's Diagnostic Questionnaire; YIAT, Young Internet Addiction Test.

## DISCUSSION

The potential for harm related to the internet and social media is widely discussed in both the lay and research literature, but it remains difficult to determine, which aspects may be harmful and conversely, which may be supportive. Gaming Disorder in the 11th edition of the *International Classification of Diseases*[27] and Internet Gaming Disorder in the fifth edition of the *Diagnostic and Statistical Manual of Mental Disorders* (DSM), are potential diagnoses requiring further research [28], and the importance of the ‘internet’ aspect is not clear. Internet Addiction, also referred to as problematic or pathological internet use, dominated the findings of this systematic review, but the addiction model of internet use does not account for more nuanced behavioural mechanisms, which we shall now discuss.

### Mechanisms of harm

There is growing evidence of specific mechanisms by which social media/internet use can be harmful, especially in relation to young people. In a recent meta-analysis, cybervictimization was associated with suicide attempts (OR 2.57, 95% CI 1.69–3.90) [11^▪▪^]. Furthermore, a link was reported between cyberbullying, suicidal ideation and self-harm, highlighting it as an extremely important area of focus when considering social media/internet risks.

Exposure to social media/internet has the potential to both suggest and reinforce negative thoughts and behaviours. There is an association between comments on Instagram with increasing severity of self-injury, suggesting social media may act to reinforce harmful behaviours [29]. Themes such as self-loathing, loneliness and feeling unloved were found in content analysis of 3360 randomly selected Tumblr posts from 17 depression-related accounts; 82% of posts were related to depression, suicide or self-harm [30]. There are differences in how social media platforms are used, for example, different trends in image posting between Twitter and Tumblr or Instagram [31^▪▪^]. Understanding the functions across online platforms that are supportive or detrimental for different age groups, or populations, will be important to guide clinicians’ line of enquiry, risk assessment as well as recommendations about social media/internet use and future interventions.

The internet provides an unrivalled opportunity to access information but sometimes the ideas gleaned can be detrimental for vulnerable young people. Capacity (dispositional, acquired and practical) to make a suicide attempt is an important factor in the Three-Step Theory of suicide [32] and it is not yet known if, or how, social media/internet could impact this. The Darknet is an under-researched entity, with potentially significant implications for risk assessment of suicidal youth because of the anonymity conferred by not being indexed by conventional search engines. Morch *et al.*[33] found fewer websites devoted to suicide on the Darknet via the Tor browser (4%), compared with the Surface Web (23.1%) [34] but this appears to be the only study of its kind. Violent methods are associated with increased risk of suicide [35^▪^], and the internet, especially the Darknet, has the potential to increase availability to harmful means. Determining, which young people access different levels of web content and whether access to the Darknet is associated with more violent or effective means of suicide could be important areas of future research.

Internet search trend data has the potential for wide reaching possibilities in terms of surveillance and detection of those at risk of suicide. Chandler[36] found a positive correlation between search intensity of suicide-related terms and the number of suicides across America between 2006 and 2014, particularly for youth. This methodology has also been replicated in the UK [37], but in both cases, not specifically looking at adolescent populations. This may suggest an association between suicide internet search activity and suicide. However, the usefulness of search trends on a population level to understand risk, especially for specific groups, such as adolescents, is limited at present.

With the rise of social media/internet, there is concern about the potential for electronic communication to facilitate clusters of youth suicides [38]. Following a potential suicide cluster in the United States, online social networking was identified as having broad relevance, both positive and negative, on how young people hear about a suicide, the impact on them, their perceptions of the environment afterwards and recovery [39]. In response to this potential risk, some research has generated evidence-based guidelines to help youth discuss suicide-related themes safely on social media [40^▪^], though more research is required to understand this complex phenomenon.

### Support and a force for good

Frost *et al.* found one-third of young people with a history of self-injurious behaviour had used the internet to seek help in relation to self-harm. Over half of these online help-seekers perceived that they had more support available to them online than offline [41]. In their analysis of depression-related accounts on Tumblr, Cavazos-Rehg *et al.*[30] found that 9% of posts involved direct interaction with others and of these, 47% provided emotional support or reassuring messages.

Intensive community treatment can reduce need for hospital admission among adolescents [42] and there is increasing evidence that both ‘self-driven’ and ‘socially-driven’ processes can decrease suicide attempts [43]. Youth-Nominated Support Team Interventions (which include youth-nominated caring adults) have potential to reduce mortality in suicidal adolescents [44]. No intervention studies have been done using social media for suicide prevention, but there are examples of social media sites designed for suicide prevention, including sites with potential to reach those at risk of suicide [45], building on the increasing self-harm and suicide intervention evidence base.

Writing style could help detect suicidal youth via online platforms through identification of: internal attribution, excessive self-focus and higher psychological pain and cognitive constriction [46]. Social media content on self-harm is not always used to actively encourage others to self-harm, but predominantly to express difficult emotions and inspire recovery [31^▪▪^]. For some young people, the anonymous potential of social media/internet may make it an easier place to express themselves and find support, beyond what can be offered via conventional means.

### Strengths and limitations of this review

We did not include studies with participants 19 years or over, or which referred to ‘suicidal behaviour’ where suicide or suicide attempts were not specified. These narrow criteria, as well as exclusion of grey literature and non-English language publications may have excluded relevant studies. Further, the heterogeneity of exposure and outcome measures made synthesizing evidence challenging and prevented the combination of studies in a meta-analysis. However, the specific nature of our question has highlighted the existing literature on suicide, suicide attempts and social media/internet use and will be relevant to child and adolescent clinicians, as well as highlighting areas of interest for future study.

## CONCLUSION

Current evidence suggests that excessive or ‘problematic’ use of social media/internet does impact suicide risk, specifically increasing the risk of suicide attempts. Longitudinal studies are vital to establish the direction of the potential association, the impacts of potential confounders, such as sleep disturbance and cyberbullying and recommendations on safe amounts of use. As internet and social media platforms develop, more understanding of the specific risks and mechanisms associated with different types of digital activity, by different population groups will be essential to understand risk and pave the way for specific interventions.

## Acknowledgements

None.

### Financial support and sponsorship

R.S. and S.E. have been employed as National Institute of Health Research (NIHR) Academic Clinical Fellows. S.E. also received salary support from an MQ Data Science Award and from the Psychiatry Research Trust. R.D. is supported by a Clinician Scientist Fellowship from the Health Foundation in partnership with the Academy of Medical Sciences. D.O. is supported by research grants with the Medical Research Council (grant mr/r004927/1) and the National Institute for Health Research.

The authors acknowledge infrastructure support from the National Institute for Health Research (NIHR). The views expressed are those of the authors and not necessarily those of the NHS, the NIHR or the Department of Health.

### Conflicts of interest

There are no conflicts of interest.

## REFERENCES AND RECOMMENDED READING

Papers of particular interest, published within the annual period of review, have been highlighted as:

▪ of special interest
▪▪ of outstanding interest
